# Assessment of Genetical, Pre, Peri and Post Natal Risk Factors of Deciduous Molar Hypomineralization (DMH), Hypomineralized Second Primary Molar (HSPM) and Molar Incisor Hypomineralization (MIH): A Narrative Review

**DOI:** 10.3390/children8060432

**Published:** 2021-05-21

**Authors:** Andrea Butera, Carolina Maiorani, Annalaura Morandini, Manuela Simonini, Stefania Morittu, Stefania Barbieri, Ambra Bruni, Antonia Sinesi, Maria Ricci, Julia Trombini, Elisa Aina, Daniela Piloni, Barbara Fusaro, Arianna Colnaghi, Elisa Pepe, Roberta Cimarossa, Andrea Scribante

**Affiliations:** 1 Unit of Dental Hygiene, Section of Dentistry, Department of Clinical, Surgical, Diagnostic and Pediatric Sciences, University of Pavia, 27100 Pavia, Italy; carolinamaiorani@outlook.it; 2 Member Association: “ Mamme & Igieniste”, 24125 Bergamo, Italy; dr.annalauramorandini@gmail.com (A.M.); manuelasimonini@libero.it (M.S.); morittustefania3@gmail.com (S.M.); stefaniabarbieri@me.com (S.B.); ambrabruni@libero.it (A.B.); antonia.sinesi@gmail.com (A.S.); maridental70@gmail.com (M.R.); juliatrombini@hotmail.it (J.T.); elisa.aina@libero.it (E.A.); piloni.daniela@libero.it (D.P.); bfusaro@libero.it (B.F.); colnaghi.arianna@gmail.com (A.C.); elipepe82@gmail.com (E.P.); roberta.id@cimarossa.it (R.C.); 3 Unit of Orthodontics and Pediatric Dentistry, Section of Dentistry, Department of Clinical, Surgical, Diagnostic and Pediatric Sciences, University of Pavia, 27100 Pavia, Italy; andrea.scribante@unipv.it

**Keywords:** deciduous molar hypomineralization, hypomineralization second primary molar, molar incisor hypomineralization, pediatric dentistry, dental hygienist

## Abstract

Objectives: Analyze defects in the state of maturation of the enamel result in an adequate volume of enamel, but in an insufficient mineralization, which can affect both deciduous teeth and permanent teeth. Among the most common defects, we recognize Deciduous Molar Hypominerlization (DMH), Hypomineralized Second Primary Molar (HSPM), and Molar Incisor Hypomineralization (MIH). These, in fact, affect the first deciduous molars, the second deciduous molars and molars, and permanent incisors, respectively, but their etiology remains unclear. The objective of the paper is to review studies that focus on investigating possible associations between genetic factors or prenatal, perinatal, and postnatal causes and these enamel defects. Materials and methods: A comprehensive and bibliometric search for publications until January 2021 was conducted. The research question was formulated following the Population, Intervention, Comparison, Outcome strategy. Case-control, cross-sectional, cohort studies, and clinical trials investigating genetic and environmental etiological factors of enamel defects were included. Results: Twenty-five articles are included. For genetic factors, there is a statistical relevance for SNPs expressed in the secretion or maturation stage of amelogenesis (16% of studies and 80% of studies that investigated these factors). For prenatal, perinatal, and postnatal causes, there is a statistical relevance for postnatal factors, such as the breastfeeding period (2%), asthma (16%), high fever episodes (20%), infections/illnesses (20%), chickenpox (12%), antibiotic intake (8%), diarrhea (4%), and pneumonia (4%). Conclusions: The results are in agreement with the multifactorial idea of the dental enamel defects etiology, but to prove this, further studies enrolling larger, well-diagnosed, and different ethnic populations are necessary to expand the investigation of the genetic and environmental factors that might influence the occurrence of DMH, HPSM, and MIH.

## 1. Introduction

Dental enamel is a highly mineralized tissue consisting of 95% of hydroxyapatite crystals arranged into highly ordered units called enamel prism, which creates a structure with remarkable mechanical resistance. During development, ameloblasts produce and secrete the organic component, and then they polarize and are removed from the surface of the enamel. Precisely for this reason, the mature enamel does not have the capacity of regeneration, due to not having any more living supporting cells [1,2].

Generally, a fragile and quantitatively defective enamel or a normal volume enamel but with insufficient mineralization can be found, depending on which phase of the ameloblasts life cycle is affected [3]. In this case, if we speak of insufficient mineralization, we can include enamel defects such as Deciduous Molar Hypomineralization (DMH), Hypomineralized Second Primary Molar (HPSM), and Molar Incisor Hypomineralization (MIH). DMH is defined as an enamel defect of systemic and multifactorial origin that affects the second deciduous molar [4]; its prevalence varies between 4.9% and 9.0% [5]. HSPM is described as demarcated qualitative defects of systemic origin enamel affecting one or more second primary molars [6]; its prevalence varies between 0% and 21.8% [7]. MIH is characterized by demarcated qualitative defects of enamel of systemic origin affecting one or more first permanent molars with or without incisor involvement [8]; its prevalence varies between 2.9% and 4.4% [7].

Compared to healthy teeth, teeth affected by enamel defects show a less distinct histologically prism sheath, with a lack of hydroxyapatite crystals: the hypomineralized enamel has lower mechanical properties, such as hardness and elasticity, which have lower values than a normal enamel. In addition, the enamel affected by these pathologies shows a greater amount of proteins such as serum albumin, type I collagen, ameloblastin, a1-antitrypsin, and antitrobin III, which inhibit the growth of the hydroxyapatite crystals, resulting in the reduction of the enamel minerals [9].

The risk of onset of these lesions appears to be affected by several factors related to prenatal, perinatal, and postnatal phases, such as: severe illness or complications during pregnancy, low birth weight or premature birth, problems related to breastfeeding or to illness, infections in the first years of life of the child. However, defects in the development of dental enamel can be caused not only by environmental factors but also by genetic disorders [10]. In fact, amelogenesis and odontogenesis are processes that are subject to genetic control. There is, however, little information on this subject, or on the influence of genetic variations in the genes coding the proteins of the enamel matrix: factors affecting ameloblastes and influencing the mineralization of the enamel [11].

The etiology of enamel defects, particularly of the highly discussed MIH, remains unclear to this day [11], despite the fact that environmental and genetic factors, as well as factors related to the prenatal, perinatal, and postnatal period, seem to play an important role [12]. The objective of this review is to investigate the possible associations between genetic, but also prenatal, perinatal, and postnatal factors and these enamel defects.

## 2. Material and Methods

### 2.1. Focused Question

Which genetic factors and problems related to the prenatal, perinatal, and postnatal period cause enamel defects like Deciduous Molar Hypomineralization (DMH), Hypomineralized Second Primary Molar (HPSM) and Molar Incisor Hypomineralization (MIH)? In particular, can they influence the development of MIH?

### 2.2. Elegibility Criteria

First, we analyzed the studies in accordance with the following inclusion criteria:

Type of studies. Case-control, cross-sectional, cohort studies and clinical trials, properly registered, with the approval of the Ethics Committee;

Type of participants. Participants with Deciduous Molar Hypomineralization (DMH)/Hypomineralized Second Primary Molar (HPSM)/Molar Incisor Hypomineralization (MIH) were selected in a range from pregnancy up to 28 years old.

Type of interventions. Case-control, cross-sectional, cohort studies, and clinical trials that have evaluated the possible etiological factors involved in the development of enamel defects.

Outcome type. Each variable included in the studies was taken into account; we included studies that have assessed the possible association between the problematic factors (prenatal, perinatal, postnatal, and genetic factors). Primary outcomes: genetic factors, if these have been taken into account by the studies. Secondary outcomes: complications/illness/infections, use of antibiotics/medicines, intake of folic acid, calcium or fluoride, the use of cigarettes or alcohol intake during pregnancy (prenatal factors); birth weight, childbirth (for example premature birth or caesarean section), hypoxia, respiratory disorders, Apgar Score (perinatal factors); breastfeeding, illness/disorders/infections, allergies, asthma, high fever episodes, intake of calcium or fluoride (postnatal factors).

We included in the second phase only those studies that met all the inclusion criteria, that is to say, the analysis of the selected studies according to the exclusion criteria (I) studies where the authors had not reported at least one of the parameters chosen as outcomes; (II) studies performed on participants with concomitant systemic pathologies/treatments that could have affected outcomes; (III) studies that have not analyzed the possible causes of enamel defects; (IV) in vitro or animal clinical studies; (V) studies carried out without the approval of the Ethics Committee.

### 2.3. Search Strategy

The review is based on the research of studies in reference to the PICO model (Population, Intervention, Comparison, Outcome), identified through bibliographic research in electronic databases, and by examining the bibliography of articles, on Pubmed and MEDLINE. Initially, all study abstracts that evaluated the possible factors involved in the development of dental enamel defects were taken into consideration.

### 2.4. Research

We performed the search using the following keywords: “dental enamel diseases”, “deciduous molar hypomineralization”, “hypomineralized second primary molar”, “molar incisor hypomineralization”, “etiology of dental enamel diseases” OR “etiology of deciduous molar hypomineralization”, OR “etiology of hypomineralized second primary molar”, OR “etiology of molar incisor hypomineralization”, “deciduous molar hypomineralization” AND “genetic factors”, OR “deciduous molar hypomineralization” AND “prenatal factors”, OR “deciduous molar hypomineralization” AND “perinatal factors”, “deciduous molar hypomineralization” AND “postnatal factors”, OR “hypomineralized second primary molar” AND “genetic factors”, OR “hypomineralization second primary molar” AND “prenatal factors”, OR “hypomineralized second primary molar” AND “perinatal factors”, OR “hypomineralized second primary molar” AND “postnatal factors”, OR “molar incisor hypomineralization” AND “genetic factors”, OR “molar incisor hypomineralization” AND “prenatal factors”, OR “molar incisor hypomineralization” AND “perinatal factors”, OR “molar incisor hypomineralization” AND “postnatal factors”. There was no time limit on the date of publication of the study.

### 2.5. Screening and Selection of Articles

**Figure 1:**
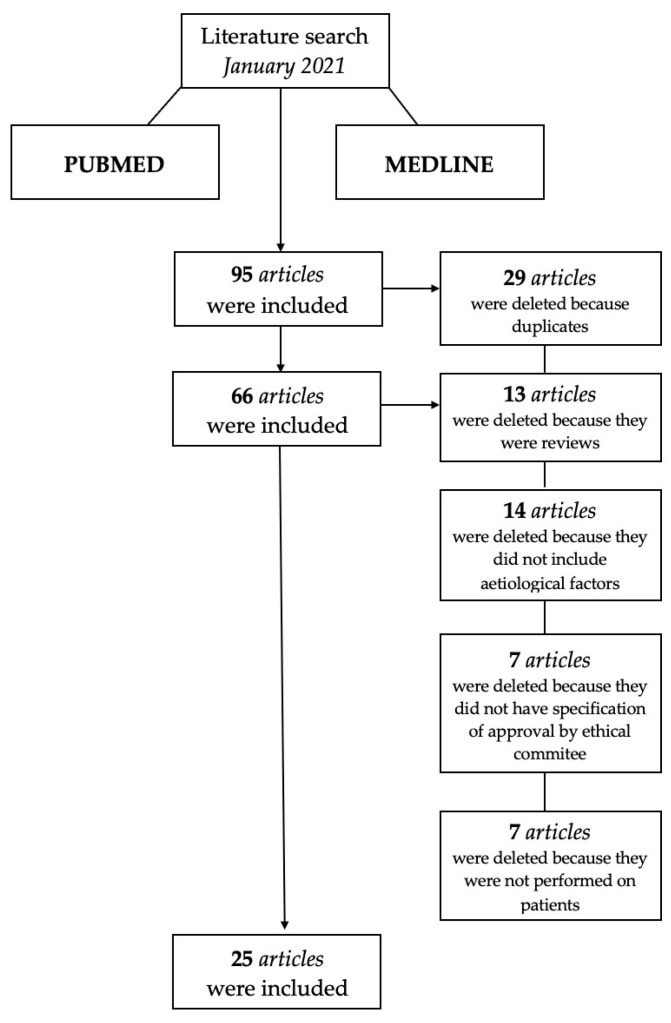
Flow chart of included studies.

The search produced 95 titles matching the search keywords and the information related to the inclusion criteria. The following flowchart shows the selection criteria used to select the final 25 articles that were used for the review analysis, see Figure 1.

### 2.6. Search Outcome and Evaluation

The first research outcomes are genetic factors. Other interesting outcomes that were present are prenatal, perinatal, and postnatal factors. Information was extracted from each study on (I) participants’ characteristics (age and disease characteristics) and criteria for inclusion and exclusion from the studies in question; (II) intervention (modality); (III) outcome (possible associations between dental enamel defects and etiological factors); (IV) data examined (genetic and environmental factors); (V) other data (less significant or less considered).

## 3. Results

### 3.1. Study Selection

Ninety-five articles on the etiological factors of dental enamel defects emerged from several researchers. After a first reading of the abstracts found, the following were discarded: (I) articles that emerged in more researches carried out; (II) reviews and meta-analysis; (III) studies where the authors did not report at least one of the parameters chosen as outcomes; (III) studies performed on participants with concomitant systemic pathologies/treatments that could have affected outcomes; (IV) studies that did not analyze the possible causes of enamel defects; (V) in vitro or animal clinical studies; (VI) studies carried out without the approval of the Ethics Committee.

A total of 25 studies were therefore identified: 5 studies on the possible genetic factors involved; 15 studies on the possible pre-, peri-, postnatal factors involved; 2 studies on possible pre- and postnatal factors involved; 1 study on possible pre- and perinatal factors involved; 1 study on possible prenatal factors involved. Table S1 shows the selected studies according to the PICO model.

### 3.2. Synthesis of Results

#### 3.2.1. MIH (Sixteen Studies)

*Methods*. The 16 studies selected for the review were published in English: 31.25% of the studies are cross-sectional studies, 25% are case-control studies, 18.75% are cohort studies, and the remaining 25% are also divided between retrospective, longitudinal, observations, and clinical trials. Twenty-five percent were conducted in Brazil, 12.5% in Turkey and the remaining 62.5% are divided between Ethiopia, Lebanon, Saudi Arabia, France, Germany, Colombia, Italy, Slovenia, Iraq, and USA.

*Participants*. Studies on average recruited about 917 patients (917, 43), where 62.5% of the studies included less than that amount of patients, and 37.5% of the studies included more than 917 patients. 

*Intervention*. Patients were selected to assess the possible association between MIH and the etiological factors involved in this defect: 68.75% of studies investigated the possible pre-, per-, post-natal factors involved, 25% of studies investigated the possible genetic factors involved, and the other 6.25% investigated the possible peri-natal factors involved. Studies were performed using a natural light or mirrors to determine the presence/absence of MIH; a questionnaire was also given to parents in most of these studies. 

*Outcome*. The aim of the study was to investigate the possible association between MIH and genetic or pre-, peri-, and post- natal factors [13,14,15,16,17,18,19,20,21,22,23,24,25,26,27,28]. 

#### 3.2.2. DMH (Two Studies)

*Methods*. The two studies selected for the review are cohort studies conducted in the Netherlands and in Switzerland, published in English. 

*Participants*. A total of 6690 patients aged 6 years were enlisted for each study.

*Intervention*. Patients were selected to assess the possible association between DMH and the etiological factors involved in this defect: the study was performed using a natural light and mirrors to determine the presence/absence of DMH; a questionnaire was also given to the parents in these studies as well. 

*Outcome*. The study’s outcome was to investigate the possible association between DMH and pre-, peri-, and post- natal factors [5,29]. 

#### 3.2.3. HPSM (Two Studies)

*Methods*. The two studies selected for review are a cross-sectional one conducted in Brazil and a cohort one conducted in Australia, both published in English.

*Participants*. The cross-sectional study recruited 811 patients of 5 years of age, while the cohort study recruited 172 twins; the average was about 576 patients per study. 

*Intervention*. Patients were selected to assess the possible association between HPSM and the etiological factors involved in this defect: the study conducted in Brazil was performed to analyze, on four primary fully erupted second molars, the association between HPSM and pre-, peri-, and postnatal factors, as well as the study conducted on twins; studies were performed using light and mirrors to determine the presence/absence of HPSM; a questionnaire was also given to the parents.

*Outcome*. The aim of the study was to investigate the possible association between HPSM and pre-, peri-, and post- natal factors [30,31].

#### 3.2.4. MIH and DMH (One Study)

Methods. One study selected for the review is a longitudinal study published in English; this was conducted in Brazil. 

*Participants*. A total of 134 children aged between 4 and 6 years participated in the study. 

*Intervention*. Patients were selected to assess the possible association between DMH and MIH and the etiological factors involved in both defects: the study was performed using a natural light and mirrors to determine the presence/absence of MIH/DMH; a questionnaire was also given to parents. 

*Outcome*. The study’s outcome was to investigate the possible association between MIH/DMH and pre-, peri-, and post- natal factors [32].

#### 3.2.5. MIH and HPSM (Three Studies)

*Methods*. The three studies selected for the review are two cross-sectional studies and one cohort study published in English; one study was conducted in Brazil, while the other two were carried out in the Netherlands. Randomization of the studies was performed with different methods: only one by computer-generated sequence and the others by generation R studies. 

*Participants*. Studies recruited on average about 3027 patients aged between 6 and 8 years; 66.7% of studies included less than 3027 patients, and 33.3% of studies included more than 3027 patients. 

*Intervention*. Patients were selected to assess their dental enamel status in relation to possible genetic or environmental factors: one study was performed to analyze four erupted first molars and the association between dental enamel defects and polymorphisms in vitamin D (*rs 739837 and rs 2228570*) on four erupted first molars, while the others were conducted to evaluate the association between dental enamel defects and pre- and post- natal factors, such as serum 25(OH)D concentrations and bone mass. Photographs were taken in two studies to assess the presence or absence of MIH/HPSM, and in one study data collection was performed using artificial light and a dental mirror; also, in one of the studies, a questionnaire was given to the parents. 

*Outcomes*. Most of the studies investigated the possible association between MIH/HSPM and pre- and post- natal factors; only one study was focused on the possible role of vitamin D polymorphism in the development of MIH/HPSM [33,34,35].

#### 3.2.6. Genetic Factors

Possible genetic causes related to MIH development were discussed in five of the analyzed studies (20%); one study evaluated this possible correlation with MIH and HPSM. The studies involved have the following objectives:

To investigate the genetic carriage potentially involved in MIH development. In this study, significant results were obtained for SNPs *rs 7821494*, *rs 34367704*, rs 3789334, *rs 60994896*, *rs 762642*, *rs 7664896*, *rs 1711399*, *rs 1711423*, *rs 2278163*, *rs 6996321*, and *rs 5979393*; so variations in genes related to amelogenesis were associated with the susceptibility to develop MIH [13].

To investigate the relationship between MIH and possible genetic loci: in this study, *rs 13058467*, that is located near the SCUBE1 gene on chromosome 22, was identified as a possible locus linked to MIH [20].

To investigate some genetic factors potentially involved in MIH development: in this study, SNP *rs 2245803* in the MMP20 gene in a homozygous form in a recessive model was associated with MIH development [25].

To investigate whether polymorphism in vitamin D receptor genes increases the prevalence of MIH and HPSM: in this study, no association between MIH and HPSM with *rs 739837* and *rs 2228570* polymorphisms was found [33]. 

To investigate the interaction of the interferon regulatory factor 6 (IRF6) and the transforming growth factor alpha in predisposition of MIH (TGFA). In this study, a potential interaction between TGFA rs930655 with all markers tested in one cohort was found [28].

These results were in conformity with multifactorial ideas of the HPSM/MIH etiology and the potential genetic factors involved.

#### 3.2.7. Pre-, Peri-, Post-Natal Factors

Possible pre-, peri-, post-natal factors related to DMH/HPSM/MIH development were discussed in 20 studies of those analyzed (80%): 60% of these studies investigated pre-, peri-, post-natal factors, while 8% of these investigated pre- and post-natal factors, and the remaining 12% investigated pre-natal factors/peri-natal factors or pre- and peri-natal factors.

Pre-natal factors. By comparing the results, the following factors were identified as significant in the development of this type of injury: problems/complications in pregnancy (20% of studies; this condition is correlated to the development of MIH) [14,15,17,21,27], alcohol consumption or smoking (8% of studies; this condition is correlated to the development of MIH and HPSM) [31,34] and the intake of antibiotics (4% of studies; this condition is correlated to the development of MIH) [23].

Peri-natal factors. By comparing the results, among the most significant peri-natal causes, the following were identified as significant factors in the development of this type of injury: premature childbirth (12% of studies; this condition is correlated to the development of MIH and HPSM) [14,25,30], type of childbirth (12% of studies; this condition is correlated to the development of MIH) [15,19,21], hypoxia or respiratory problems (12% of studies; this condition is correlated to the development of MIH) [19,21,27], birth weight (8% of studies; this condition is correlated to the development of MIH and DMH) [27,29], and intake of vitamin D (4% of studies; this condition is correlated to the development of HPSM) [31].

Post-natal factors. Breastfeeding period (8% of studies; this condition is correlated to the development of MIH) [14,27], asthma (16% of studies; this condition is correlated to the development of MIH and HPSM) [14,16,18,30], high fever episodes (20% of studies; this condition is correlated to the development of MIH and DMH) [14,18,24,26,27], infections/illness (20% of studies; this condition is correlated to the development of MIH) [14,15,18,22,27], chickenpox (12% of studies; this condition is correlated to development of MIH) [14,18,24], antibiotic intake (8% of studies; this condition is correlated to the development of MIH) [18,23], diarrhea (4% of studies; this condition is correlated to the development of MIH) [14], and pneumonia (4% of studies; this condition is correlated to the development of MIH) [24].

Table S2 shows the pre, peri-, and post-natal factors most extensively analyzed in the studies.

#### 3.2.8. Results of Single Studies and Bias

**Figure 2:**
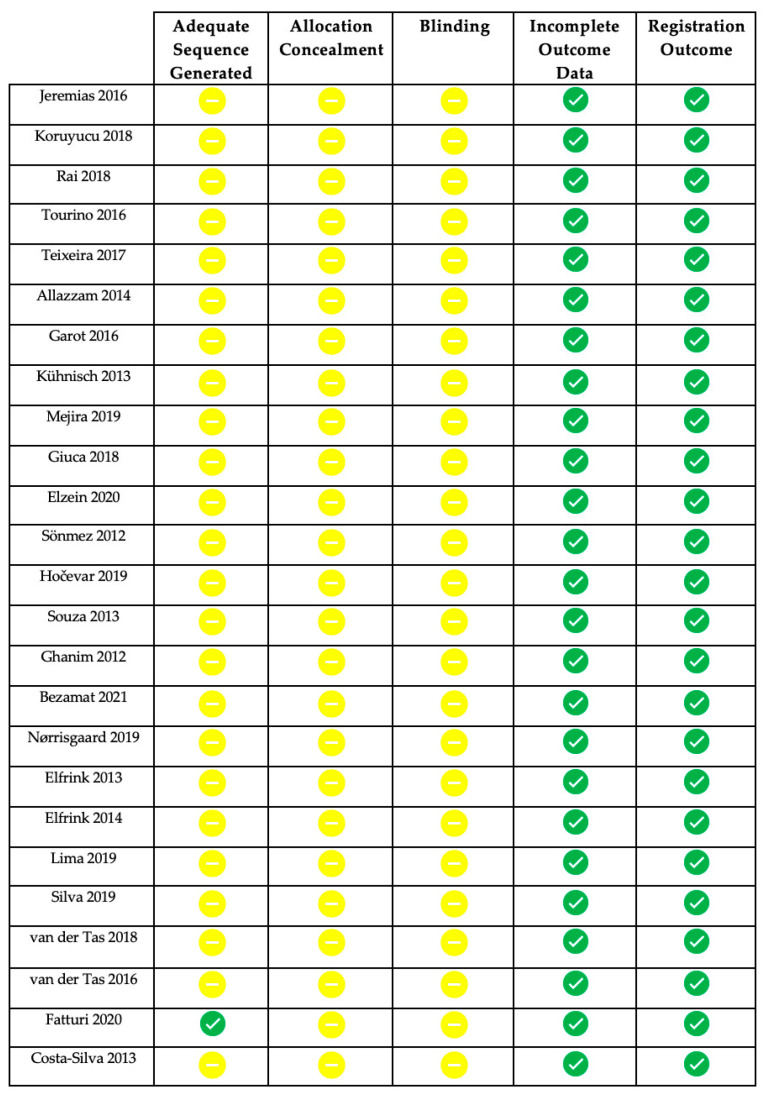
Green symbol: low risk of bias; yellow symbol: moderate risk of bias (also used for lack of information).

The risk of bias has been assessed according to the type of randomization and the allocation concealment, the blinding, the outcome data, and the registration of the outcomes on the basis of the information described in the articles (for example, concerning randomization sequence generation, a low bias risk was assessed for studies using a random numbering table or a computer random number generator). Risk of bias could not be assessed due to insufficient detail; in the absence of information, a moderate risk was considered. Figure 2 shows the risk of bias of the main articles examined; this review presents a relatively moderate risk of bias.

## 4. Discussion

There are many factors that could affect amelogenesis and result in DMH, HPSM, and MIH, so the purpose of this review is to investigate which environmental and genetic factors were the most involved in the development of enamel defects, such as Deciduous Molar Hypomineralization (DMH), Hypomineralized Second Primary Molar (HPSM), and Molar Incisor Hypomineralization (MIH). The clinical presentation of localized and asymmetrical lesions suggests a systemic origin, dependent on the amelogenesis process in the early maturation stage or even earlier at the late secretory phase. 

From the emerged results, the multifactorial etiology of these pathologies becomes even more known, in accordance with several studies in the literature: in fact, any type of environmental or genetic factor potentially involved in the development of injuries to the dental enamel has been the subject of observation; many studies, however, have never reached exhaustive conclusions, noting a lack of strong statistical relevance of the factors studied [36].

Multiple possible causes have been suggested in the literature, for instance, the possibility of a genetic role in the etiology, indicating that a genetic variation may interact with systemic factors leading to dental enamel defects; this is in accordance with the results obtained in this review. In fact, we found that variations in genes related to amelogenesis were associated with the susceptibility to develop MIH: SNPs *rs 7821494*, *rs 34367704*, *rs 3789334, rs 60994896*, *rs 762642*, *rs 7664896*, *rs 1711399*, *rs 1711423*, *rs 2278163*, *rs 6996321*, and *rs 5979393*. This last gene is fundamental for amelogenesis, which codifies amelogenin, the main protein of dental enamel secreted by ameloblasts during the secretion stage of amelogenesis [13,37,38]; but the other ones, like *rs 7664896* and *rs 34367704,* expressed in the maturation stage of amelogenesis, are also implicated [13,37]. At the same time, it affects dental enamel defects, prenatal, perinatal, and postnatal factors [38,39,40,41,42], such as respiratory tract infections, perinatal complications, hypoxia, low birth weight, calcium metabolic disorders, childhood diseases, use of antibiotics/drug, and prolonged breastfeeding. In this case, we found the most statistical relevance for postnatal factors, such as the breastfeeding period, asthma, high fever episodes, childhood illness or infections (oral, ear, throat, respiratory, urinary infections), diarrhea and childhood diseases like chickenpox, renal failure, rubella, parotitis, adenoids and tonsillitis, eczema, otitis. Those were investigated in some of the studies [14,16,18,23,24,27,30].

Although in fewer numbers, we also found some relevance in pre- and perinatal causes, such as the use of antibiotics in pregnancy, alcohol consumption or smoking and complications related to the last trimester of pregnancy [14,15,17,21,23,27,31,34], as they can affect the type of childbirth, premature birth, birth weight, the Agar Score, and respiratory problems [14,15,19,21,25,27,29,30,31]. 

Therefore, if a certain cause is not yet identifiable, it is essential to focus on every possible risk factor most closely involved in making early diagnosis and formulating an equally timely treatment. The management of this type of enamel is rather complex, both for the clinical aspect and for the need for treatment: it ranges from the prevention of the rupture of the enamel, to the management of hypersensitivity or pain, up to the extraction of the element concerned.

The decision on the choice of treatment must be made individually, taking into account the severity of the lesions, the symptomatology of the affected tooth, the age, and the aesthetic expectations of the patient [43]. The therapy must therefore include prevention, recovery, immediate treatment, and long-term planning; the following points are of particular importance in the prophylaxis program for low mineralized areas: regular professional oral hygiene, use of fluoride preparations (topical fluoride prophylaxis or three/four applications of sodium fluoride or fluoride varnish), and the use of CPP-ACP products and sealings [44,45]; it is also useful to use biomimetic nano-hydroxyapatite for the reduction of enamel hypomineralization. This has the greatest effectiveness if applied daily for a minimum of 2–3 min up to a maximum of 10 min for 10 days a month [46].

Table 1 concerning the possible causes, clinical signs and possible therapies to be proposed to the patient, depending on the severity of the defect.

However, if the damage is more complex, it will be necessary to carry out a composite restoration, in the case of MIH of mild and moderate lesions, or steel crowns and indirect ceramic restorations, in the case of serious defects [40]. Appropriate knowledge of all the possible factors involved in the development of enamel defects is needed to develop effective prevention protocols or implement targeted treatment protocols, thus avoiding possible enamel fractures, hypersensitivity to hot or cold foods and aesthetic problems in the area of the front teeth: treatment of teeth affected by MIH should be a minimally invasive procedure that aims to protect, strengthen and preserve dental structure; in the context of minimally invasive therapy, several remineralizing products based on biomimetic hydroxyapatite [46] or infiltrating resins [47] are available, thus it is possible to manage the symptoms resulting from the hypomineralization of the dental enamel.

**Table 1:** Etiological factors, clinical signs, and possible therapy.

Etiological Factors	Clinical Signs	Possible Therapy
SNPs *rs 7821494*, *rs 34367704*, *rs 3789334*, *rs 60994896*, *rs 762642*, *rs 7664896*, *rs 1711399*, *rs 1711423*, *rs 2278163*, *rs 6996321* and *rs 5979393* Antibiotics in pregnancy, alcohol consumption or smoking and complications related to the last trimester of pregnancy Breastfeeding period, asthma, high fever episodes, childhood illness or infections (oral, ear, throat, respiratory, urinary infections), diarrhea and childhood diseases, such as chickenpox, renal failure, rubella, parotitis, adenoids and tonsillitis, eczema, otitis.	Mild: The demarcated opacities located at non-stress bearing areas, no caries associated with the affected enamel, no hypersensitivity, and incisor involvement is usually mild if present; Moderate: The demarcated opacities present on molars and incisors, the post-eruptive enamel breakdown limited to one or two surfaces without cuspal involvement, atypical restorations can be needed and normal dental sensitivity; Severe: Post-eruptive enamel breakdown, crown destruction, caries associated with affected enamel, a history of dental sensitivity and aesthetic concerns [48].	Regular oral hygiene sessions with professional dental cleaning Use of fluoride preparations (local fluoridation) Use of CPP-ACP products and sealings. If the damage is greater and requires restoration, there are several options, such as: Composite restorations in case of mild to moderate defects (anchorage in healthy enamel), Steel crowns as a long-term in the case of severe defects (subsequent reconstruction with lab-produced restorations), and Indirect restorations (fabricated in the laboratory or with CAD/CAM) made of composite or ceramic.

## 5. Conclusions

These results are in compliance with the multifactorial idea of the etiology of dental enamel defects.

The maturation period of the tooth enamel that is affected by enamel defects corresponds to the last trimester of pregnancy to the third year of a child’s life, and it is possible that a genetic variation can in some way interact with environmental factors: considering the latter, hypoventilation in many respiratory diseases such as asthma can cause respiratory acidosis and abnormal oxygen levels, which affect the pH of the enamel matrix: these hinder the action of proteolytic enzymes and development of hydroxyapatite crystals; furthermore, considering that ameloblasts derive from the epithelium, the opacities can represent irreparable scarring of the enamel following degenerative changes caused by chickenpox, which is known to attack the epithelial surfaces.

## Data Availability

The data presented in this study are available on request from the corresponding author.

## Supplementary Materials

The following are available online at https://www.mdpi.com/article/10.3390/children8060432/s1, Table S1: Included studies concerning DMH, HPSM, MIH; Table S2: Pre, Peri and Post Natal Causes.
