# Supplementary material for: Assessment of Genetical, Pre, Peri and Post Natal Risk Factors of Deciduous Molar Hypomineralization (DMH), Hypomineralized Second Primary Molar (HSPM) and Molar Incisor Hypomineralization (MIH): A Narrative Review
Source: Children (Basel). 2021 May 21;8(6):432. doi: 10.3390/children8060432 (PMC8224286; doi:10.3390/children8060432)
Supplement: Supplementary file 1 [file children-08-00432-s001.zip › children-1228707-supplementary.pdf]

Supplementary

Table S1. Included Studies Concerning DMH, HPSM, MIH.

Title	Authors	Year	Evidence	P (Problem)	I (Intervention)	C (Comparison)	O (Outcome)	Investigated pre-natal factors	Investigated peri-natal factors	Investigated post-natal factors	Investigated genetic factors
**Investigation of clinical characteristics and etiological factors in children with molar incisor hypomineralization**	Giuca MR, Cappè M, Carli E, Lardani L, Pasini M	**2018**	Case-control	60 children with MIH and 60 children without MIH aged from 6 to 13 years, Caucasian and with the presence of at least one permanent molar with MIH with or without incisors involved (fot the thest group) were included in this study. The purpose of this was to evaluate the clinical defects and etiological factors potentially involved in the onset of MIH.	Test group: clinical examination of MIH was performed on wet teeth after cleaning. A questionnaire was distributed to parents in order to investigate the possible etiological factors of MIH (prenatal, perinatal, postnatal). Control group: A questionnaire was distributed to parents in order to investigate the possible etiological factors of MIH (prenatal, perinatal, postnatal)		A total of 186 molars and 98 incisors exhibited MIH defects: 55 molars and 75 incisors showed mild defects, 91 molars and 20 incisors had moderate lesions and 40 molars and 3 incisors showed severe lesions. Univariate and multivariare statistical analysis showed a significat association between MIH and ear, nose, and throat disorders and the antibiotics used during pregnancy.	Fluoride intake during pregnancy; gestational diabetes, taking drugs or smoking.	Childbirth complications.	Breastfeeding; allergies; penicillin use; vitamin D; infections; ENT disorders; respiratory disorders	None
**Pre and postnatal determinants of deciduos molar**	Elfrink MEC, Moll HA,	**2014**	Cohort	Assessments were planned in early, mid and late	Children with DMH (5183): children visited the research		A number of factors in the pre-, peri- and postnatal	Additional use folic acid; maternal	Twin pregnancy;	Breastfeeding (6 months), additional vitamin D,	None

**hypomineralisation in 6 year-old children. The generation R study.**	Kiefte-de-Jong J, Jaddoe VWV, Hofman A, ten Cate JM, Veerkamp JSJ			pregnancy and included questionnaires on lifestyle and general health, physical examinations and fetal ultrasound examinations. Postnatal information on the growth, development and health of the participating children at the ages of 2, 6 and 12 months was obtained from manual measurements at the routine child health centres and by questionnaires.	centre for manual measurements and to have photographs taken of their teeth. Children without DMH: children visited the research centre for manual measurements and to have photographs taken of their teeth.	phase were found to be associated with DMH. After multivariate logistic regression analyses, Dutch ethnic background, low birth weight, maternal alcohol consumption during pregnancy, and fever episodes in the first year of the child's life were found to play a role in the developement of DMH in 6-year-old children.	alcohol consumption during pregnancy; air pollution; medication use mother; illness; vomitting and diarrhea; pregnancy induced diabetes; high bloodpressure because of pregnancy	birh weight ; apgar score; hospit alisati on 1st week of life	medication use mother during breastfeeding; medication use child; fever episodes; illness; shortness of/breath/wheezing; vomitting and diarrhea	
**Molar incisor hypomineralization: prevalence and risk factors among 7-9 years old school children in Muradnagar, Ghaziabad**	Rai A, Singh A, Menon I, Singh J, Rai V, Singh Aswal G	**2018**	Cross-sectional	A total of 992 school children (7-9 years old) with all first permanent molars and incisors erupted were examined to investigate the prevalence and risk factors of permanent MIH.	The study proforma was divided into 2 parts, first part coverage general information that comprise of demographic status and socio-economic status, questionnaires on risk factors of MIH such as prenatal, perinatal and postnatal history	The overall prevalence rate of MIH was 21,4% in this study. Age, problems during pregnancy, normal delivery and childhood illness/infections are the risk factors wich have highest strenght of association.	Gestational diabetes; hypertension; hypocalcaemia; vitamin D deficiency.	Birth compli cations ; birth prema turity; type of birth	Childhood illness and infections; otitis/ear infections; asthma; chicken pox; allergies; antibiotics use; breastfeeding.	None

					upto 3 years. The second part comprised of recording format of MIH and dental caries by using DMFT index.					
**Influence of deciduos molar hypomineralization on the development of molar-incisor hypomineralization**	Costa-Silva CM, Simpson de Paula J, Bovi Ambrosano GM, Mialhe FL	**2013**	Longitudinal	134 children aged 4 to 6 years were evaluated six-montly until they completed the eruption of the first permanent molars to invistigate propspectively the association between DMH and MIH and the etiologic factors involved in both defects.	In order to observe the presence of DHM and subsequently the MIH, the surfaces of the teeth were examined without drying after monitored brushing. Only children who were in the deciduous dentition phase were included in the survey. All secondary primary molars were examined for demarcated enamel opacity and the chldren were prospectively evaluated for two years by six-monthly evaluation until they completed the eruption of the first permanent molars. The possible factors involved in the etiology of	It could be noted that, although most children with DMH in the primary dentition had higher incidence of MIH in permanent dentition, this difference was not statistically significant.	None	Birth weight; type of delivery	None	None

					hypomineralized enamel defects were reviewed to produce a questionnaire. Then, 4 groups were formed: children with DMH and MIH, children with MIH and without DMH, children with DMH and without MIH, children with MIH and no DMH.					
**Peripartum events and molar-incisor hypomineralisation amongst young patients in southwest France**	Garot E, Manton D, Rouas P	**2016**	Retrospective	This study, carried out between 2010 and 2011, was based on objective information noted in child health booklets on putative risk factors for MIH during the Peripartum period, aged between 6 to 28 years. The aim was to investigate the association of peripartum events with the occurrence of MIH.	Among the 849 patients examined by two calibrated paediatric dentists, 75 patients with MIH were recorded. The control group was chosen randomly (from 6 to 27 years of age, from the same facilities) are included. Patients who did not possess their personal health record or if notes for the period of interest were not recorded were also excluded from this study.	Correlations were observed between hypoxia during delivery and occurance of MIH and also between birth by caesarean section and MIH. there was no association between prematurity and MIH.	None	Type of childbirth; apgar score; birth weight	None	None
**Molar incisor hypomineralization in Colombia: prevalence, severity**	Mejira JD, Restrepo M, Gonzales	**2019**	Observational	1075 children were included to determine prevalence, severity	Schoolchildren between 6 and 15 years of age, born in the city of Medellin,	Prevalance of MIH was 11,2% and the majority of defects were mild. MIH	Maternal illness	Type of childbirth;	Fever; urinary infections; antibiotics use; breastfeeding	None

**and associated risk factors**	S, Alvarez LG, Santos-Pinto L, Escobar A		and risk factors associated with MIH in schoolchildren between 6 and 15 years of age in Colombia. A questionnaire addressing gestational period and first three years of child's life was administred to biological numbers. Two calibrated examiners established MIH diagnosis.	with fully erupted first molars and permanent incisors, complete etiological factors assessment questionnaire, and signed informed consent were included in this study. Schoolchildren between 6 and 15 years of age, born in the city of Medellin, with fully erupted first molars and permanent incisors, complete etiological factors assessment questionnaire, and signed informed consent were included in this study. Questionnaires filled out by adoptive parents, legal guardians (non-biological), or other family members were excluded from the analysis in order to guarantee trustworthy answers from the biological mother regarding pregnancy and	was associated with alterations during last gestational trimester, type of childbirth and respiratory problems.		apgar score; birth weight	

					child's first three years of life.				
**Molar incisor hypomineralization, prevalence, and etiology**	Allazzam SM, Alaki SM, El Meligy OAS	**2014**	Cross-sectional	A group of 8-12 years old children with at least one first permanent molar erupted or partially erupted were recruited to evaluate the prevalence and possible etiological factors associated with MIH.	A questionnair was carrefully constructed to identify all possible etiological conditions associated with MIH and releated to the child or parental history, specially the mother. The questionnaire asked about demographic data, maternal health and medicatons intake during pregnancy, feeding practices and child's medial history during the first four years of life.	MIH showed a prevalence of 8,6%. Demarcated opacities were the most common form. Maxillary central incisors were more affected than mandibular. The condition was more prevalent in children with history illness during the first years of life including tonsillitis, adenoiditis, asthma, fever and antibiotics intake.	Illness during pregnancy; medications	Type childbi rth; birth weight	breastfeeding; adenoiditis; fever; tonsillitis; asthma	None
**Exploring the association between genetic and environmental factors and molar incisor hypomineralization: evidence from a twin study**	Barbosa Teixeira RJP, Andrade NS, Carvalho Cavalcant e Queiroz L, Medeiros Mendes F, Moura MS, De Deus	**2017**	Cross-sectional	167 pairs of twins were selected to evaluated the agreement of MIH between monozygotic and dizygotic twin pairs and the association with enviromental factors.	The parents answered a questionnaire on sociodemographic data and pre-, peri- and postnatal health. A dental examination was performed by two calibrated examiners for MIH diagnosis, after supervised oral hygiene with a dental brush and	The prevalence of MIH was 29,3%. There was a greater concordance of MIH between monozyotic twins for affected first molars and permanent incisors and pairs of twins assessed with family income between one and	Fever during pregnancy; diseases during pregnancy	Type of childbi rth; birth weight ; hypoxi a; respira tory distres s	Fever; otitis; antibiotics use	None

	Moura LDFA, Deus Moura Lima M				fluoridated toothpaste.	two wages, above two wages and gestational hemorrhage.				
**Association between molar incisor hypomineralization in schoolchildren and both prenatl and postnatal factors: a population-based study**	Fonseca Padua Goncavales Tourino L, Correa-Faria P, Conceicao Ferreira R, Baccin Bando C, Zarzar PM, Pimenta Vale M	**2016**	Cross-sectional	1181 schoolchildren were recruited to evaluate the prevalence of MIH and identify associated prenatal, perinatal and postnatal factors among Brazilian schoolchildren aged 8 an 9 years.	Information on demographic and socioeconomic characteristics as well as prenatal, perinatal and postnatal aspects was obtained throught questionnaires. The clinical examination included the investigation on MIH; dental caries in the permanent dentition and developmental defects of enamel on the primary second molars were also recorder.	The prevalence of MIH was 20,4%. MIH was more frequent among children with dental caries in the permanent dentition, those with DDE on the primary second molars and those who experienced asthma/bronchitis in the first four years of life.	Drugs during pregnanacy	Type of childbirth	Breastfeeding; pneumonia; asthma/bronchitis; antibiotics or analgesics use; fever	None
**The relationship between molar incisor hypomineralization, dental caries, socioeconomi factors, and polymorphism in the vitamin D receptor gene: a**	Lopes Fatturi A, Menoncin BL, Torres Reyes M, Meger M, Scariot R, Brancher JA, Calvano Kuchler E,	**2020**	Cross-sectional	731 schoolchildren were selected to investigate whether polymorphisms in vitamin D receptor genes increase the prevalence of dental caries, MIH and HPSM	MIH, HPSM and dental caries were clinically assessed by four calibrated examiners using EAPD criteria, DDE index and DMFT index. A structured questionnaire was completed by the children's caregivers	Schoolchildren with MIH presented a higher prevalence of dental caries. No association was observed between MIH, HPSM, and dental caries, with *rs739837* and *rs2228570*	None	None	None	Polymorphism in the vitamin D receptor gene

**population-based study**	Feltrin-Souza J				to collect socioeconomic data from the population.	polymorphisms. Individuals with the GT/GG genotype in *rs739837* polymorphism presented a higher prevalence of MIH in molars and incisors than indivisuals TT.				
**Prevalence and etiology of molar-incisor hypomineralization (MIH) in the city of Istanbul**	Koruyucu M, Ozel S, Bahar Tuna E	**2018**	Longitudinal	1511 8- to 11-year-old children were examined who had their first permanent molar and incisors to assess the prevalence and the risk factors of MIH in children in Istanbul	Hypomineralized molars and incisors were recorded based on developmental defects of enamel index. The potential aetiological factors were retrieved through personal interview and etiological questions were asked to the parents.	MIH was observed in 215 children. The sample (1511 children) comprised 71 (9.9%) 8 year-olds with MIH and 144 (18.2%) 11 year-olds with MIH. A significant difference was found between 8 (9.9%) and 11-year-old (18.2%) children with MIH ($p < 0.001$). Complications during the mother's pregnancy, birth prematurity, average breast feeding period, diarrhea frequency,	Complications during pregnancy	Birth weight; type of childbirth	Breastfeeding; diarrhea; asthma; pneumonia; respiratory diseases; throat infections; fever; ear infections; renal failure; urinary infections; parotits; rubeola	None

						digestive system diseases, asthma, frequent high fever, ear infection, renal failure, rubeola, chickenpox and parotitis were found to be significantly associated with MIH (p <0.001).				
**Molar-incisor hypomineralisation in Lebanon: association with prenatal, perinatal and postnatal factors**	Elzein R, Chouery E, Abdel-Sater F, Bacho R, Ayoub F	**2020**	Case-control	659 schoolchildren aged 7-9 year-old were selected to investigate the association of MIH with prenatal, natal and postnatal factors	The partecipants were first examined for the diagnosis of MIH using the MIH index, if they having one or more FPM was affected with or without the involvement of the incisors and when at least one of the incisors was erupted. A self-administred, structured questionnaire consistin in 19 close-ended questions was distributed to children's parents.	Children whose mothers had consumed food and drinks canned during pregnancy had 2.9 times more likely to have MIH. Those who had history of taking antibiotics had 2.15 times higher odds of MIH than those who did not have while those who had fever episodes during the early childhood years were 2.057 times more likely to develop MIH.	Medical problems	Type of childbi rth	Otits; fever; allergies; asthma; pneumonia; antibiotics use; breastfeeding	None
**Pre-term birth and asthma is associated with hypomineralizaed**	Rodrigues Silva Lima L, Souza Pereira A,	**2020**	Cross-sectional	811 pre-schoolers with 5 years old were selected in Teresina (brazil) to	Sociodemographic status and pre-, peri-, and post-natal conditions were	The prevalence of HSPM was 14.9. Demarcated opacities (75.6%)	Fever/infection s during regnancy; urinary	Hypox ia; type of childbi	Pneumonia; asthma; sinusitis; fever; otitis; antibiotics use	None

**second primary molars in pre-schoolers: a population-based study**	Silva de moura M, Castelo Branco Lima C, Paiva SM, de Fatima Almeida de Deus Moura L, de Deus Moura de Lima M			determine the prevalence and factors associated with HSPM. The sample was randomly selected and stratified by geographic region of the city, type of school and sex.	collected by structured questionnaires. Two calibrated examiners diagnosed HSPM using the criteria of the EAPD for molar-incisor hypomineralization. Severity, colour, location, and extent of lesions were also evaluated.	and white/cream colour were the most prevalent (71.4%). Pre-term pre-schoolers had 66% and those who reported asthma in the first year of life had 69% higher prevalence of HSPM. Pre-term birth and reported asthma in the first year of life were associated with HSPM. The prevalence of HSPM in pre-schoolers aged 5 years old in Teresina was high.	infections during pregnancy; antibiotics use	rth; respiratory difficulty at birth; birthweight		
**Genome-wide association study (GWAS) for molar-incisor hypomineralization (MIH)**	Kuhnisch J, Thiering E, Heitmuller D, Tiesler CMT, Grallert H, Heinrich-Weltzien R, Hickel R, Heinrich J	**2013**	Cohort	Clinical and genetic data from the 10-year follow-up of 668 children were assessed to investigate the relationship between MIH and possible genetic loci.	After recruiting newborns in Munich, follow-up visits with the children were conducted at the ages of 6 months, 1 year, 18 months, 2, 4, 6 and 10 years. The dental examinations included the diagnosis of MIH according to the criteria of the EAPD. Children with MIH were categorized as	A total of 2,013,491 single-nucleotide polymorphisms (SNPs) were available for analysis. Rs13058467, which is located near the SCUBE1 gene on chromosome 22 (p<3.72E-7), was identified as a possible locus linked to MIH when using a	None	None	None	Genetic loci

					those with a minimum of one hypomineralized first permanent molar. A GWAS was implemented following a quality-control step and an additive genetic effect was assumed.	threshold of p value <1E-6.				
**Association between bone mass and dental hypomineralization**	van der Tas JT, Elfrink MEC, Vucic S, Heppe DHM, Weerkamp JSJ, Jaddoe VWV, Rivadeneira F, Horman A, Moll HA, Wolvius EB	**2016**	Cross-sectional	6150 children aged 6 year-old, starting from the fetal life until adulthood, were selected to examine the association between the bone mass and HSPMs and MIH	EAPD criteria were used to score the introral photographs on the presence or absence of HSPMs and MIH; bone mass was measured with a dual-energy x-ray absorptiometry scan. Maternal age, pregnancy lenght and weight, ethnicity, income, educational level, and calcium intake were assessed via questionnaire at recruirment.	In the fully adjusted model, children with lower BMC were more likely to have HSPMs; a lower BMC was not associated with MIH. A negative association between BMC and HSPMs was observed. No association was found between BMC and MIH.			Bone mass	
**Aetiology of molar-incisor hypomineralization (mih) in Brazilian children**	Souza JF, Jeremias F, Costa-Silva CM, Santos-Pinto L, Zuanon ACC,	**2013**		1151 children aged 7-12 years were selected to determine the potential aetiological factors related to MIH in Brazil	Children were examined by two examiners evaluating the presence of MIH according to criteria suggested by EAPD. Their mothers completed a	The prevalence of MIH in the children was 12,3%. the interviewing response rate was 90,4%. The prevalence of	Risks during pregnancy; systemic diseases; medicines use; smoking	Birth weight	Breastfeeding; systemic diseases; throat infections; oral infections; antibiotics use; fever; allergies	None

	Cordeiro RCL				structured questionnaire about medical history , from pregnancy to the first 3 years of the children's life.	miscarriage history and occurence of anaemia were higher in mothers from MIH group than those from non MIH group. However, these associations were not statistically significant. In the childrn's medical history, rhinitis, bronchitis, and high fever were more prevalent in MIH group, but there were no significant differences between the groups.				
**Etiology of hypomineralized second primary molars: a prospective twin study**	Silva MJ, Kilpatrick NM, Craig JM, Manton DJ, Leong P, Burgner D, Scurrah KJ	**2019**	Cohort	250 children from twin pregnancies were recruited antenatally to investigate the relative contribution of genes and environment to the etiology of HSPM and to identify potential environmental risk factors in a	Detailed demographic, health, and phenotypic data were collected at recruitment, 24- and 36-wl gestation, birth, and 18 mo of age, 25-Hydroxyvitamin D was quantified for mothers ar 28-wk gestation and infants at birth. Dental examinations were conducted on the	Vitamin D levels at birth, infantile eczema, dizygosity, in vitro fertilization, socioeconomic position, and maternal smoking beyond the first trimester of pregnancy demonstrated the strongest associations with	BMI; infections during pregnancy; antibiotics use; vitamin D; smoking; alcohol consumption;	Birth weight ; vitami n D	Breastfeeding; infections; eczema; asthma	None

				longitudinal twin cohort.	twins ar 6 y of age to determine the presence, severity, and extent of HSPM per standardized criteria.	HSPM. Overall concordance for HSPM was 0,47 with weak evidence or higher concordance in MZ twins as compared with DZ twins. After adjusting for known risk factors, there was no evidence for an additive genetic influence.				
**Foetal, neonatal and child vitamin D status and enamel hypomineralization**	van der Tas J, Elfrink MEC, Heijboer AC, Rivadeneira F, Jaddoe VWV, Tiemeier H, Schoufour JD, Moll HA, Ongkosuwito EM, Wolvius EB, Voortman T	**2018**	Cohort	1840 partecipants have completed this study to study whether vitamin D status during foetal, postnatal and childhood periodos in associated with the presence of HSPMs and/or MIH at the age of six.	HSPMs and MIH were scored from intraoral photographs of the children at their age of six. Serum 25(OH)D concentrations were measured at three points in time, wich resulted in three different samples; mid-gestational in mothers' blood, in umbilical cord bloos and in children's blood at the age of six.	After adjustment for confounders, no association was found between foetal 25(OH)D concentrations and the presence of HSPMs or MIH in 6-year-olds. A higher 25(OH)D concentration in umbilical cord blood resulted in neither lower odds of having HSPM nor lower odd of having MIH by the age of six. Higher 25(OH)D concentrations at the age of six didn't associated	BMI; smoking; alcohol consumption; calcium intake	None	Breastfeeding	None

					with a significant change in the odds of having HSPM or MIH.				
**Is maternal use of medicine during pregnancy associated with deciduos molar hypomineralisation in the offsping? A prospective, population-based study**	Elfrink MEC, Moll HA, Kiefte-de Jong JC, El Marroun H, Jaddoe VWV, Hofman A, Stricker BH, ten Cate JM, Veerkamp JSJ	**2013**	Cohort	6690 partecipant from fetal life until young adulthood were studied to investigate thether antibacterials and antiallergic and antiashtma medicines are associated with DMH and, if so, which specific medicines.	Assesment included questionnaires, physical examinations and fetal ultrasound examinations and were planned in early, mid and late pregnancy. Data on maternal medicine use during pregnancy were retrieved from pharmacy records. Clinical photographs of the second primary molars, which were scored for DMH were taken with an intra-oral camera.	During pregnancy, 20.3% of the mothers used antibacterials, 12.3% anti-asthma medicines and 5.4% anti-allergic medicines. The prevalence of DMH was 9.0% in the study group. There was no association between the use of anti-asthma medicines, anti-allergic or antibacterials during pregnancy and DMH. The study had sufficient power to detect significant associations.	Use of antibacterial, amoxicillin, tetracycline, anti-asthma, anti-allergic; intake folic acid; illness; fever; diabetes	Apgar score	Fever; illness; fluoride intake	None
**Family-based genetic association for molar-incisor hypomineralization**	Jeremias F, Pierri RAG, Souza JF, Fragelli CMB, Restrepo M, Finoti	**2016**	Cohort	391 individuals were selected to investigate the genetic carriage potentially involved in MIH developement	Sixty-three single nucleotide polymorphisms (SNPs) were investigated in 21 candidate genes related to amelogenesis using	Significant results were obtained for SNPs rs7821494, rs34367704, rs3789334, rs6099486, rs762642, rs7664896,	None	None	None	Genetic carriage

	LS, Bussaneli DG, Cordeiro RCL, Secolin R, Maurer-Morelli CV, Scarel-Caminaga RM, Santos-Pinto L				the TaqMan TM OpenArray TM Genotyping platform. All SNPs were genotyped in 165 birth family members unaffected by MIH, 96 with unknown MIH status and 130 affected individuals (50.7% with severe MIH). Clinical examinations were performed with the use of a flashlight and a mouth mirror.	rs1711399, rs1711423, rs2278163, rs6996321 and rs5979395.				
**The possible influence of genetic aetiological factors on molar-incisor hypomineralisation**	Hocevar L, Kovac J, Trebusak Podkrajsek K, Battelino S, Pavlic A	**2020**	Cohort	113 patients who were surgically treated at an Otorhinolaryngology and Cervicofacial Surgery Clinic during childhood were selected to search the possible associations between some genetic factors that could affect the development of MIH.	HLA DQ2 and DQ8 haplotypes and single nucleotide (SNP) of eight amelogenesis-related genes were searched in genomic DNA. Genotypes were determined by high resolutin melting, TaqMan genotyping assays, and Sanger sequencing.	Among the evaluated genetic variants, SNP rs2245803 in the MMP20 gene in homozygous form in a recessive model was associated with MIH development with the genotype distribution of TT(3), TG(6) or GG(13) in children with MIH and distribution of TT(18), TG(42) or GG(31) in children without MIH.	None	None	None	Genetic loci

**Association of High-Dose Vitamin D Supplementation During Pregnancy With the Risk of Enamel Defects in Offspring: A 6-Year Follow-up of a Randomized Clinical Trial**	Nørrisgaard PE, Haubek D, Kühnisch J, Chawes BL, Stokholm J, Bønnelykke K, Bisgaard H	**2019**	Trial	623 women recruited at 24 weeks of pregnancy and 588 of their children were selected to assess the association of a high-dose vitamin D supplementation in pregnant women with enamel defects and caries in their offspring.	High-dose vitamin D3 (2400 IU/d; N = 315) or matching placebo tablets (N = 308) from pregnancy week 24 to 1 week post partum. In addition, all women received 400 IU/d of vitamin D3 as part of standard care.	The risk of enamel defects in the permanent dentition was lower in the offspring of mothers who received high-dose vitamin D supplementation during pregnancy compared with standard dose 15.1% vs 27.5%. A similar association was observed for the deciduous dentition vs 15.9%. There was no association between supplementation and caries.	High-dose vitamin D	None	None	None
**Risk factors in the occurrence of molar–incisor hypomineralization amongst a group of Iraqi children**	Ghanim A, Manton D, Bailey D, Marino R, Morgan M	**2012**	Cohort	Seven- to nine-year-old school children were recruited to inevstigate the risk actors ivolved n the development of MIH n a group of school-aged Iraqi children.	A questionnaire was used to determine the possible systemic risk factors. Information on the child's history of orofacial trauma or infection related to the primary teeth and family history of enamel defects was also obtained. The interviewer also	For children with MIH, 6% reported no relevant medical history; the remaining 94% reported various medical conditions putatively associated with MIH compared with 70% for the non-affected	Pregnancy illness	Birth weight ;hypoxia; hypocalcaemia; respiratory distress	Ear infections; urinary tract infections; fever; diarrhea; vomitting; chickenpox; tonsillitis; pneumonia; breastfeeding; use of antibiotic	None

					asked about information relating to mother's employment status at the time of pregnancy. First permanent molar and incisor teeth were examined for the presence of MIH using the EAPD evaluation criteria.	group. Post-natal medical conditions (33.3%) were most frequently reported. When data were split into the possible risk effect groups, maternal psychological stress, frequent exposure to ultrasonic scans during the last gestational trimester and birth order as a fourth sibling or later were previously unreported significant risk factors and postulated as contributing to, or causing the defect.				
**Gene-environment interaction in molarincisor hypomineralization**	Bezamat M, Souza JF, Silva FMF, Corrêa EG, Fatturi AL, Brancher JA,	**2021**	Cohort	In this present study, it was hypothesized that these genes interact and contribute to predisposition of MIH. Environmental factors affecting children that were 3 years of age or older	A total of 1,065 salivary samples from four different cohorts were obtained, and DNA was extracted from each sample and genotyped for nine different single	A potential interaction between TGFA rs930655 with all markers tested in the cohort from Turkey was identified. These interactions were not identified in	None	None	None	Genetic loci

	Carvalho FM, Cavallari T, Bertolazo L, Machado-Souza C, Koruyucu M, Bayram M, Racic A, Harrison BM, Sweat YY, Letra A, Studen-Pavlovich D, Seymen F, Amendt B, Werneck RI, Costa MC, Modesto A, Vieira AR.			were also hypothesized to play a role in the disease etiology. Those factors included respiratory issues, malnutrition, food intolerance, infection of any sort and medication intake.	nucleotide polymorphisms.	the remaining cohorts. Associations (p<0.05) between the use of medication after three years of age and MIH were also found, suggesting that conditions acquired at the age children start to socialize might contribute to the development of MIH.				
**Putative factors associated with molar incisor hypomineralisation: an epedimiological study**	Sönmez H, Yıldırım G, Bezgin T	**2013**	Case-control	4,049 children were recruited to examine the aetiological factors involved in the development of molar incisor	Putative aetiological factors were evaluated using a questionnaire sent to children’s families. The questionnaire included questions	The aetiology of MIH is not clear yet, and the results of this study support the results of previous studies regarding	Illness during pregnancy	Birth weight ; prema turuty	Breastfeeding; fever; otitis; urinary infections; respiratory infections; fluoride or calcium intake	None

hypomineralisation (MIH).	on prenatal, perinatal, and postnatal systemic conditions. Teeth were examined wet, as suggested by the FDI Working Group (Commission on Oral Health 1992) using a mirror and periodontal probe to measure the diameter of lesions	the putative causal effect of several factors.

Table S2. Pre, Peri and Post Natal Causes.

No association (% of studies)	% of studies regarding risk factor	Post-natal facotrs	Association (% of studies)	No association (% of studies)	% of studies regarding risk factor
12%	40%	Breastfeeding	8%	4%	52%
12%	40%	Diarrhea	4%	/	12%
/	16% (hypoxia); 12% (respiratory problems)	Asthma	16%	4%	28%
/	40%	Fever	20%	8%	44%
/	4%	Infections/illness	20%	4%	64%
		Chickenpox	12%	/	12%
		Use of antibiotics	8%	8%	40%
		Pneumonia	4%	4%	24%
